# Daily Dietary Intake Patterns Improve after Visiting a Food Pantry among Food-Insecure Rural Midwestern Adults

**DOI:** 10.3390/nu10050583

**Published:** 2018-05-09

**Authors:** Breanne N. Wright, Regan L. Bailey, Bruce A. Craig, Richard D. Mattes, Lacey McCormack, Suzanne Stluka, Lisa Franzen-Castle, Becky Henne, Donna Mehrle, Dan Remley, Heather A. Eicher-Miller

**Affiliations:** 1 Department of Nutrition Science, Purdue University, West Lafayette, IN 47907, USA; wrigh197@purdue.edu (B.N.W.); reganbailey@purdue.edu (R.L.B.); mattes@purdue.edu (R.D.M.); 2 Department of Statistics, Purdue University, West Lafayette, IN 47907, USA; bacraig@purdue.edu; 3 Health and Nutritional Sciences, South Dakota State University, Brookings, SD 57007, USA; lacey.mccormack@sdstate.edu; 4 Extension, South Dakota State University, Brookings, SD 57007, USA; suzanne.stluka@sdstate.edu; 5 Nutrition and Health Sciences, University of Nebraska-Lincoln, Lincoln, NE 68583, USA; lfranzen2@unl.edu; 6 Extension, Michigan State University, Charlotte, MI 48813, USA; henner@msu.edu; 7 Extension, University of Missouri, Columbia, MO 65212, USA; mehrled@missouri.edu; 8 Extension, Ohio State University, Piketon, OH 45661, USA; remley.4@osu.edu

**Keywords:** emergency food assistance, food pantry, food insecurity, dietary patterns, dietary quality

## Abstract

Emergency food pantries provide food at no cost to low-resource populations. The purpose of this study was to evaluate single-day dietary intake patterns before and after visiting a food pantry among food-secure and food-insecure pantry clients. This observational cohort study comprised a paired, before-and-after design with a pantry visit as the intervention. Participants (*n* = 455) completed a demographic and food security assessment, and two 24-h dietary recalls. Adult food security was measured using the U.S. Household Food Security Survey Module. Dietary intake patterns were assessed using Automated Self-Administered 24-h Recall data and classified by Healthy Eating Index (HEI-2010) scores, dietary variety, number of eating occasions, and energy intake. Paired *t*-tests and Wilcoxon signed-rank tests compared outcomes before and after a pantry visit. Mean dietary variety increased after the pantry visit among both food-secure (*p* = 0.02) and food-insecure (*p* < 0.0001) pantry clients. Mean energy intake (*p* = 0.0003), number of eating occasions (*p* = 0.004), and HEI-2010 component scores for total fruit (*p* < 0.001) and whole fruit (*p* < 0.0003) increased among food-insecure pantry clients only. A pantry visit may improve dietary intake patterns, especially among food-insecure pantry clients.

## 1. Introduction

Approximately 16 million Americans utilize emergency food pantries, most of whom (67%) are classified as food-insecure [1]. Food insecurity is characterized by reports of reduced dietary quality and variety, disrupted eating patterns, and reduced food intake [2]. Food insecurity in adults is associated with lower intake of vegetables, fruits, dairy products, vitamins A and B6, calcium, magnesium, and zinc compared to food-secure adults [3]. Food insecurity is also associated with indicators of diet-related chronic diseases, including increased rates of diabetes, hypertension and hyperlipidemia, as well as poorer physical and mental health, and quality of life [4]. These health limitations may, in turn, increase the burden of food insecurity and perpetuate this cycle. Emergency food pantries provide food resources to food-insecure individuals at no cost and with minimal requirements. Use of emergency food pantries by clients was originally regarded as a response to a temporary situation, but may be increasingly used on a consistent basis as a dependable food source [5].

The nutritional contributions of food pantries to client diets is largely unknown [6]. Yet, it has been estimated that food pantries could be responsible for up to 25% of the household food supply among pantry users [6]. The impact of pantry foods on client diets may also vary based on food security status. There may be two distinct groups of emergency food pantry users; one group who relies on pantries because of a short-term or “emergency” change in their economic situation (indicating food insecurity), and another group who uses pantry resources for an extended period of time as one component of their ongoing food supply (as a buffer to retain food security) [7]. Consequently, the relationship between food insecurity and dietary intake patterns among food pantry clients should be evaluated to determine the differential potential of food pantries as an intervention to improve dietary intake patterns for households that may be using food pantries in different capacities.

The objectives of this study were to quantify and compare the short-term dietary intake patterns before and after a pantry visit among rural, Midwestern adult food pantry clients overall and then stratified by food security status. We hypothesized that dietary intake patterns, including the Healthy Eating Index-2010 (HEI-2010) score as a measure of dietary quality, the number of eating occasions, energy intake, and dietary variety, would increase significantly from before compared with after receipt of pantry foods, particularly for food-insecure pantry clients.

## 2. Materials and Methods

### 2.1. Study Design

This observational cohort study comprised a before-and-after design with a pantry visit as the intervention. This study was part of a larger multi-state intervention, “Voices for Food”, which was administered through the Extension programs of universities in each of six states: Indiana, Michigan, Missouri, Nebraska, Ohio and South Dakota, and aimed to improve food security among rural, Midwestern food pantry clients. Four food pantries from counties defined as non-metro with poverty rates higher than 16% in 2011 [8], with Cooperative Extension presence, and without well-established food policy councils in each state were selected (totaling four food pantries per state). In each state, two of the food pantries were designated as “intervention” pantries and matched with “comparison” pantries based on several criteria, including: level of client choice, number of households served, pounds of food distributed per month, receipt of government commodity program assistance, food bank partnership, infrastructure and capacity (storage, shelving, etc.), and predominant racial/ethnic group served at the pantry.

### 2.2. Recruitment

From August to November 2014, a convenience sample of participants was recruited through flyers that advertised the study during pantry operation hours, and by approaching clients while they waited in line to receive food at selected pantries. Participants were screened by a trained interviewer. Only clients who were English speaking, adults ≥18 years (or ≥19 years in Nebraska where the legal age criteria classifying adult status is 19 years), who visited this food pantry at least one time prior to recruitment, and who were receiving foods from the pantry on the day of recruitment were invited to participate. The South Dakota State University and Ohio State University Institutional Review Boards approved research activities prior to beginning the study and participants gave consent before completing study materials. A sample size goal of 78 pantry clients in each food security subgroup was sought based on a meaningful change in HEI total score from a previous study [9], and estimates of correlation and standard deviation of the paired sample using pilot study data.

### 2.3. Participants

A total of 613 pantry clients were confirmed eligible and recruited. Four hundred and seventy-four (77%) participants completed two single-day 24-h dietary recalls. However, because of incomplete dietary and food security data, only 455 (74%) participants were included in the final analysis. Significant differences were found between pantry clients who completed multiple recalls compared to pantry clients who completed the initial recall only; significant differences were noted only for state (*p* < 0.0001) and soup kitchen use (*p* = 0.005; data not shown).

### 2.4. Instruments

The initial interview was conducted at the pantry by trained research staff in a semi-private area. Participants completed an electronic or paper version of a questionnaire that elicited information on demographic and pantry use characteristics, and included the validated 18-item U.S. Household Food Security Survey Module (US HHFSSM) [2]. Following this questionnaire, participants completed the Automated Self-Administered 24-h Dietary Recall (ASA24™-2014), an internet-based 24-h recall [10], with optional staff assistance. An additional dietary recall was self-completed, or completed through an assisted phone interview, within two weeks of the pantry visit. Participants received $10 as compensation in the form of a grocery store gift card upon completion of the initial interview (including the questionnaire and first dietary recall), and an additional gift card for completing the second dietary recall. Sixteen percent of initial recalls and 45% of 2nd recalls captured a weekend day.

### 2.5. Data Analysis

Food security status over the past 12 months was measured using the US HFSSM. Ten of the items were used to classify food security among household adults as per previous direction [11]. A raw score (number of affirmative responses on the food security scale) of zero was categorized as high food-secure, a score of 1–2 was categorized as marginal food-secure, a score of 3–5 was categorized as low food-secure and a score of 6–10 was categorized as very low food-secure. Food security status was dichotomized into two groups: “food-secure” (included high and marginal food-secure groups) and “food-insecure” (included low and very low food-secure groups).

Dietary information from ASA24™-2014 was used to determine the single-day dietary intake patterns (including before-pantry and after-pantry single-day energy intake, HEI-2010 scores, number of eating occasions, and number of unique USDA food codes). The total number of eating occasions was determined from the self-reported intake of meals, snacks, and beverages. The number of unique food items consumed for each participant was determined using the USDA food code, a unique, eight-digit number that is assigned to identify each food and beverage item included in nutrient composition databases. The HEI-2010 is an overall measure of diet quality that indicates conformance to the Dietary Guidelines for Americans and is comprised of 12 component scores: Total Fruit, Whole Fruit, Total Vegetables, Greens and Beans, Whole Grains, Total Dairy, Total Protein, Seafood and Plant Proteins, Fatty Acids, Refined Grains, Sodium, and Empty Calories (i.e., solid fat, alcohol, and added sugars) [12]. Each of the 12 components are weighted to yield a HEI-2010 total score that has a maximum value of 100, indicating full adherence to the Dietary Guidelines for Americans (DGA), and a minimum value of 0, indicating no adherence to the DGA [12]. Because the data were collected prior to the release of the 2015 DGA and HEI-2015, the HEI-2010 was the appropriate metric to use for this study.

### 2.6. Statistical Analysis

Prevalence of participant characteristics was compared across food security status using chi-square analysis (significance *p* < 0.05). The mean number of unique USDA food codes, mean number of eating occasions, mean HEI-2010 total and component scores, and mean energy intake were estimated for the pre-pantry and post-pantry recall and compared for all clients as well as food-secure and food-insecure subgroups. Wilcoxon signed rank tests determined differences in before-pantry and after-pantry intakes for the number of unique food codes (statistically significant when *p* < 0.05) and number of eating occasions (statistically significant when *p* < 0.05/2 sub-categories of eating occasions as ‘Meals and Snacks’ and ‘Meals,’ using Bonferroni-type adjustment for multiple comparisons of sub-groups). Paired *t*-tests determined differences in before-pantry and after-pantry intakes for mean energy intake (statistically significant when *p* < 0.05) and total and component HEI-2010 scores (statistically significant when *p* < 0.05/13 HEI total and component scores, using Bonferroni-type adjustment for multiple comparisons of sub-groups). A post-hoc analysis was performed to infer whether or not improvement in dietary outcomes was a direct result of the pantry visit. The mean, median and mode of lag time were determined. Linear regression models with the response being the change in HEI total and component scores (recall 2-recall 1) and the predictors being lag time and household size were performed (statistical significance *p* < 0.05). All analyses were completed using SAS version 9.4. (SAS Institute, Hong Kong, China) and R version 2.11.1.

## 3. Results

Pantry clients in the sample were predominately white (81%), female (72%), aged 45–65 (45%), and classified as food-insecure (78%) (Table 1). When characteristics were stratified by food security status, significant differences were observed for state, age, and the number of times the pantry was visited in the last 12 months. A greater proportion of food-secure pantry clients (35%) reported being >65 years old compared to food-insecure pantry clients (16%). A greater proportion of food-secure (63%) pantry clients reported visiting the pantry six or more times compared to food-insecure pantry clients (47%).

A significant increase in mean energy intakes (before: 1400 ± 870, after: 1600 ± 880, *p* < 0.0001), mean number of eating occasions (before: 3.2 ± 1.1, after: 3.3 ± 1.1, *p* = 0.002) and mean number of unique food codes (before: 9 ± 5, after: 11 ± 5, *p* < 0.0001) was observed among all adult emergency food pantry clients from before to after the pantry visit (Table 2). However, when separated by food security status, a significant increase in the mean energy intake (before: 1400 ± 890, after: 1600 ± 890, *p* = 0.0003) and number of eating occasions (before: 3.1 ± 1.1, after: 3.3 ± 1.1, *p* = 0.004) was only noted among food-insecure food pantry clients, while a significant increase in the mean number of unique food codes was noted among both the food-secure (before: 11 ± 4, after: 12 ± 6, *p* = 0.02) and food-insecure (before: 9 ± 5, after: 11 ± 5, *p* < 0.0001) groups (Table 2). 

Despite this increased in dietary intake patterns after a pantry visit, overall dietary quality, quantified using the mean total HEI score, was poor (mean HEI-2010 total score of 41), and a statistically significant difference in HEI-2010 total score before and after a pantry visit was not observed, regardless of food security status (Table 3). A significant increase in the mean HEI-2010 total fruit (before: 1.2 ± 1.9, after: 1.7 ± 2.2, *p* < 0.0001) and whole fruit (before: 0.9 ± 1.8, after: 1.4 ± 2.1, *p* < 0.0001) scores was observed among all pantry clients. After stratifying by food security status, there was a significant increase observed only among food-insecure pantry clients for the mean total fruit (before: 1.1 ± 1.9, after: 1.7 ± 2.1, *p* < 0.001) and whole fruit (before: 0.8 ± 1.7, after: 1.3 ± 2.0, *p* = 0.0003) HEI-2010 component scores.

Post-hoc analysis showed that the average lag time was 3.7 days with both a median and mode of two days (results not shown), and lag time was inversely associated with change in Whole Fruit score (data not shown).

## 4. Discussion

Research regarding the relationship between food insecurity and dietary intake among food pantry clients is limited [13,14,15,16,17]. This study represents the first investigation of single-day dietary intake patterns before and after food pantry use for food-secure and food-insecure pantry clients. Dietary variety increased for both food-insecure and food-secure pantry clients from before compared to after visiting a pantry, while an indicator of the fruit intake component to dietary quality, energy intake, and the number of eating occasions improved only for food-insecure pantry clients.

Overall dietary quality among food pantry clients was poor, a finding that is consistent with other studies evaluating dietary quality among food pantry clients [15]. The estimated HEI-2010 total score and component scores, indicating adherence to the Dietary Guidelines for Americans, for pantry clients observed in this study were low compared to the most recent estimate among the U.S. population (59.0 ± 1.0) [18]. Component scores for total fruit, whole fruit, greens and beans, and seafood and plant protein were especially low in this group, and indicate a critical need for improvement. These results are perhaps expected considering the high prevalence of food insecurity in the sample. Seventy-eight percent of participants were classified as food-insecure. Although much higher than the U.S. population, as expected [19], the prevalence of food insecurity in this rural Midwestern food pantry-user participant sample was consistent with other studies that have evaluated food security among emergency food system clients [9,15,17,20].

Dietary quality, dietary variety, number of eating occasions, and energy intake were expected to increase significantly after receipt of pantry foods based on the premise that pantry users visit the pantry to obtain more foods. Results revealed no significant increase in overall dietary quality from before compared with after pantry use, but did reveal a significant increase in the quality of the fruit dietary component. Providing enough food (quantity) may be more of a concern to emergency food pantry providers compared with the quality of foods provided. In support of this, studies have found that food packages provided to clients by food pantries do not meet recommended nutritional requirements and may be low in fruits, dairy, whole grains and fish [21,22,23], all of which are key components of the HEI-2010 index. This may explain why the quantity of food may increase after using a pantry, while the overall quality measured by the HEI-2010 total score may remain unchanged. While lower than U.S. averages [18], component scores for total fruit and whole fruit (total fruit excluding juice) significantly increased after receipt of pantry foods. The increase in whole fruit score suggest that the increase in the total fruit component score may not be entirely due to an increase in juice intake. Although many pantries may not offer the recommended amount of fruit [21,22,23], results from this study suggest that the fruit offered by pantries is an improvement upon what clients are otherwise able to obtain, or that foods offered by pantries allow clients to use other funds to purchase fruits and represents potential for the food pantry to enhance dietary quality.

Only food-insecure pantry clients experienced a significant increase in the number of eating occasions and energy intake after visiting the pantry. Food insecurity is characterized by reports of reduced dietary quality, dietary variety, disrupted eating patterns, and reduced food intake [2], suggesting greater need for resources to restore dietary patterns. This supports the hypothesis that food-secure and food-insecure groups may use pantries differently; food-insecure pantry clients may rely on pantries in response to a dire situation, while food-secure pantry clients may use pantries continually to serve as a buffer to maintain food security. In support of this idea, the results revealed a greater prevalence of pantry use (≥6 times in the past 12 months) among food-secure pantry clients (63%) compared to food-insecure pantry clients. Therefore, food-insecure pantry clients may exhibit a higher degree of dietary restriction due to circumstance before visiting the pantry and consequently have a higher potential for improving their dietary intake patterns upon receipt of pantry foods. Both food security subgroups experienced an increase in dietary variety. Food-insecure pantry clients may receive foods from pantries that they cannot receive otherwise using non-pantry resources and therefore pantry use increases their food choices and improves dietary variety. On the other hand, food-secure pantry clients may rely on pantries consistently to acquire staple foods which they are able to supplement using other non-pantry resources, thereby improving dietary variety.

### 4.1. Strengths

Most prior studies evaluating the dietary intake of food pantry clients used only a single 24-h recall [14,15,24,25,26] with assessment completed on the day the client presented at the food pantry [15,24,25]. This study characterized the dietary patterns of pantry clients before and after visiting the pantry among a large multi-state sample of rural, Midwestern U.S. adults by assessing the dietary intake from two 24-h recalls.

### 4.2. Limitations

The observed changes in dietary intake patterns before and after pantry use may not be a direct effect of pantry use since food pantries are not the only source of foods for clients. Participants received a $10 grocery store gift card upon completion of the initial recall, which may have been used to purchase foods that clients otherwise would not have been able to afford and thus impacted dietary patterns in their second recall; however, it was unethical to withhold compensation or provide it only to participants who completed two recalls. Additionally, the research team did not assess whether or not clients visited additional pantries between the initial dietary recall and the follow-up recall, and the present study and others have reported that clients may use multiple pantries [7,27,28]. A large proportion of the secondary recalls were collected on a weekend day; previous research has indicated that diet quality is lower and energy intake is higher on weekends compared to weekdays [29], which may have biased the results. The lag time between the first and second 24-h recall could range from two days to two weeks, and it was noted that the amount of food provided by pantries is typically small. This study population had an average lag time of 3.7 days with both a median and mode of two days. Thus, in a study population where most participants reported foods lasting a few days to two weeks, application of the results is appropriate. In support of this conclusion, lag time was inversely associated with change in Whole Fruit score, suggesting that improvement in whole fruit intake decreases as time passes after visiting the pantry. Finally, because of the nature of the emergency food system, the study sample was disproportionately food-insecure and therefore there was a discrepancy in the sample sizes of the food security groups after stratification. This may have resulted in increased power for statistically significant changes in dietary intake patterns in the food-insecure group compared to the food-secure group, and thus underestimated the impact of pantry foods on diet for food-secure clients. The sample size of the present study was based on a meaningful change in HEI total score; thus, the study may not have had statistical power to detect differences in HEI component scores before and after a pantry visit, and may explain the several non-significant results. This could be improved in future studies by increasing sample size, and ultimately statistical power.

## 5. Conclusions

Food pantries may be utilized to increase dietary variety for all patrons as well as energy intake, number of meals consumed, and fruit intake specifically among food-insecure pantry clients. Food pantries may be an ideal environment for a dietary intervention to improve food security and dietary intake patterns by improving the quality, quantity, and variety of foods offered. Future research should focus on determining the usual nutrient and food group intake of food pantry clients and comparing the intake by food security status while adjusting for potential confounders in efforts to examine how pantry foods may mediate dietary intake differently among and between food-secure and food-insecure pantry clients.

## Figures and Tables

**Table 1:** Characteristics of a multistate sample of rural, Midwestern, adult emergency food pantry clients by food security status (*n* = 455).

	All Pantry Clients		Food-Secure		Food-Insecure		χ^2^
	*n*	%	*n*	%	*n*	%	*p*-Value ^1^
Total ^2^	455		100	22	355	78	
State							0.04
Indiana	117	26	23	23	94	26	
Michigan	87	19	13	13	74	21	
Missouri	102	22	21	21	81	23	
Nebraska	49	11	10	10	39	11	
Ohio	50	11	14	14	36	10	
South Dakota	50	11	19	19	31	9	
Age							0.0004
18–44 years	136	35	28	32	108	35	
45–64 years	176	45	29	33	147	48	
>65 years	81	20	31	35	50	16	
Sex							0.3
Male	107	28	28	32	79	26	
Female	280	72	59	68	221	74	
Race							0.3
White	305	81	67	78	238	82	
Black	32	8	10	12	22	8	
American Indian	28	7	8	9	20	7	
Other	12	3	1	1	11	4	
Ethnicity							0.1
Hispanic	15	4	1	1	14	5	
Not Hispanic	362	96	82	99	280	95	
Income							0.2
<$10,000	221	52	42	46	179	54	
$10,001–$15,000	91	22	26	28	65	20	
>$15,000	110	26	24	26	86	26	
Number of Pantries Visited (past 12 months)							0.1
1 pantry	203	46	50	53	153	44	
≥2 pantries	239	54	44	47	195	56	
Household Food From Food Pantry							0.2
A few days’ worth	191	45	34	40	157	46	
One to two weeks’ worth	147	35	29	34	118	35	
More than half of the food for the month	86	20	23	26	63	19	
Times Visited This Pantry (past 12 months)							0.03
0–1 times	73	16	14	12	59	17	
2–5 times	153	34	24	24	129	36	
≥6 times	229	50	62	63	167	47	

^1^ Statistical significance is *p* <0.05 for chi-square comparisons between food-secure and food-insecure adult food pantry clients. ^2^ Total numbers do not always add to sample size due to missing values; Percentages do not always add to 100 due to rounding.

**Table 2:** Comparison of before and after pantry dietary intake patterns (number of eating occasions, number of unique food codes reported consumed, energy intake and total HEI-2010 score) for all, food-secure, and food-insecure pantry clients in a multistate sample of rural, Midwestern, adult emergency food pantry clients (*n* = 455).

	**All Pantry Clients**				
	**Before-Pantry**		**After-Pantry**		
***n*** **= 455**	**Mean**	**SD**	**Mean**	**SD**	***p*-value ^1^**
Number of Eating Occasions ^2^	3.2	1.1	3.3	1.1	0.002 ^3^
Meals and Snacks	2.7	1.0	2.8	1.0	0.02 ^3^
Meals	2.2	0.8	2.3	0.8	0.03 ^3^
Number of Unique Food Codes ^2^	9	5	11	5	<0.0001 ^3^
Energy Intake (kcal) ^2^	1400	870	1600	880	<0.0001 ^4^
Total HEI Score ^2^	41	13	42	13	0.47 ^4^
	**Food-Secure**				
	**Before-Pantry**		**After-Pantry**		
***n*** **= 100**	**Mean**	**SD**	**Mean**	**SD**	***p*-value**
Number of Eating Occasions ^2^	3.4	1.0	3.5	1.0	0.2 ^3^
Meals and Snacks	3.0	0.9	3.0	0.9	0.3 ^3^
Meals	2.4	0.7	2.5	0.8	0.7 ^3^
Number of Unique Food Codes ^2^	11	4	12	6	0.02 ^3^
Energy Intake (kcal) ^2^	1500	770	1600	840	0.1 ^4^
Total HEI Score ^2^	46	13	45	14	0.4 ^4^
	**Food-Insecure**				
	**Before-Pantry**		**After-Pantry**		
***n* = 355**	**Mean**	**SD**	**Mean**	**SD**	***p-*value**
Number of Eating Occasions ^2^	3.1	1.1	3.3	1.1	0.004 ^3^
Meals and Snacks	2.6	1.0	2.7	1.0	0.04 ^3^
Meals	2.1	0.8	2.2	0.8	0.1 ^3^
Number of Unique Food Codes ^2^	9	5	11	5	<0.0001 ^3^
Energy Intake (kcal) ^2^	1400	890	1600	890	0.0003 ^4^
Total HEI Score ^2^	40	13	41	13	0.2 ^4^

^1^ Statistical significance is *p* < 0.05 for paired *t*-test and Wilcoxon signed rank test comparisons between before- and after-pantry energy intake and number of unique food codes; Statistical significance is *p* < 0.025 for paired *t*-test comparisons between before- and after-pantry number of eating occasions (*p* < 0.05/2 subcategories of ‘Meals and Snacks’ and ‘Meals’, Bonferroni-type adjustment for multiple comparisons of sub-groups); Statistical significance is *p* < 0.004 for paired *t*-test comparisons between before- and after-pantry HEI Scores (*p* < 0.05/13 HEI total and component groups, Bonferroni-type adjustment for multiple comparisons of sub-groups). ^2^ Indicates inclusion of all eating/drinking occasions: meals, snacks, and just a drink. ^3^ Indicates *p*-value was determined using the Wilcoxon signed rank test. ^4^ Indicates *p*-value was determined using the paired *t*-test.

**Table 3:** Comparison of before and after pantry HEI-2010 total and component scores in a multistate sample of rural, Midwestern, adult emergency food pantry clients for all pantry clients and for food-insecure pantry clients (*n* = 455).

		**All Pantry Clients**				
		**Before-Pantry Score**		**After-Pantry Score**		
***n*** **= 455**	**Max Score**	**Mean**	**SD**	**Mean**	**SD**	***p*-Value ^1^**
Total Vegetables	5	2.9	2.0	2.9	1.9	0.9
Green Beans	5	0.8	1.7	0.6	1.5	0.1
Total Fruit	5	1.2	1.9	1.7	2.2	<0.0001
Whole Fruit	5	0.9	1.8	1.4	2.1	<0.0001
Whole Grain	10	2.1	3.3	1.9	3.0	0.4
Total Dairy	10	4.8	3.9	5.0	3.8	0.3
Total Protein	5	3.9	1.7	4.0	1.5	0.1
Seafood and Plant Protein	5	0.9	1.7	1.0	1.8	0.3
Fatty Acid	10	4.0	3.7	4.0	3.7	0.9
Sodium	10	3.3	3.6	3.2	3.5	0.5
Refined Grain	10	6.1	3.9	6.0	3.7	0.8
Empty Calories	20	10.3	7.0	9.9	6.7	0.4
Total HEI	100	41	13.0	42	13.0	0.5
		**Food-Insecure Pantry Clients ^2^**				
		**Before-Pantry Score**		**After-Pantry Score**		
***n*** **= 355**	**Max Score**	**Mean**	**SD**	**Mean**	**SD**	***p*-Value ^1^**
Whole Fruit	5	0.8	1.7	1.3	2.0	0.0003
Total Fruit	5	1.1	1.9	1.7	2.1	<0.001
Total HEI	100	40	13	41	13	0.21

**^1^** *p*-value was determined using the paired *t*-test; Statistical significance is *p* < 0.004 for paired *t*-test comparisons between before- and after-pantry HEI Scores (*p* < 0.05/13 HEI total and components, Bonferroni-type adjustment for multiple comparisons of sub-groups). ^2^ Only HEI-2010 component scores that significantly changed from before to after a pantry visit among food-insecure pantry clients are shown.
